# Metabolomic and metagenomic analyses of the Chinese mitten crab *Eriocheir sinensis* after challenge with *Metschnikowia bicuspidata*

**DOI:** 10.3389/fmicb.2022.990737

**Published:** 2022-09-23

**Authors:** Hongbo Jiang, Jie Bao, Yuenan Xing, Gangnan Cao, Xiaodong Li, Qijun Chen

**Affiliations:** ^1^Aquaculture Department, College of Animal Science and Veterinary Medicine, Shenyang Agricultural University, Shenyang, China; ^2^Key Laboratory of Livestock Infectious Diseases in Northeast China, Ministry of Education, Shenyang Agricultural University, Shenyang, China

**Keywords:** *Metschnikowia bicuspidata*, *Eriocheir sinensis*, metabolome, metagenomic, milky disease

## Abstract

Milky disease caused by *Metschnikowia bicuspidata* fungus has significantly harmed the Chinese mitten crab *Eriocheir sinensis* aquaculture industry. However, the effect of *M. bicuspidata* infection on the metabolism and intestinal flora of the crab remains unclear. In this study, we aimed to explore the changes in the metabolism and intestinal flora *E. sinensis* after 48 h of infection with *M. bicuspidata*, using metabolomic and metagenomic analyses. Metabolomic analysis results revealed 420 significantly different metabolites between the infected and control groups, and these metabolites were enriched in 58 metabolic pathways. *M. bicuspidata* infection decreased the levels of metabolites related to amino acid biosynthesis, the tricarboxylic acid cycle, as well as lysine, histidine, linolenic, arachidonic, and linoleic acid metabolism. These results indicated that *M. bicuspidata* infection significantly affected the energy metabolism, growth, and immunity of *E. sinensis*. The results of metagenomic analysis showed that the anaerobes and ascomycetes populations significantly increased and decreased, respectively, after *M. bicuspidata* infection. These changes in intestinal flora significantly upregulated metabolic and synthetic pathways while downregulating immunity-related pathways. The results of integrated metabolomic and metagenomic analyses showed that 55 differentially expressed genes and 28 operational taxonomic units were correlated with 420 differential metabolites. Thus, the intestinal flora changes caused by *M. bicuspidata* infection also affected the metabolites. This study provides novel insights into the metabolic-and intestinal microflora-based effects of *M. bicuspidata* infection in *E. sinensis*, as well as a theoretical basis for the interaction between fungi and crustaceans.

## Introduction

**Figure 1:**
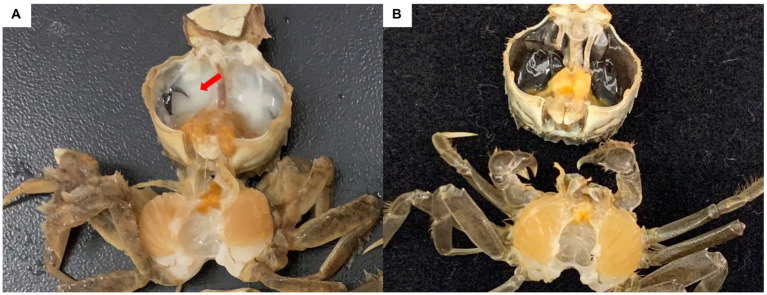
Chinese mitten crab infected with *Metschnikowia bicuspidata*. **(A)** Infected crab, with an arrow indicating the milky hemolymph. **(B)** Healthy crab.

The Chinese mitten crab *Eriocheir sinensis* is an economically important crustacean that grows in fresh water and reproduces in seawater. In 2020, the output of the Chinese mitten crab was approximately 800,000 tons, ranking first among crab types in China. In northern China, Chinese mitten crabs are primarily cultured in rice fields to help local farmers increase their incomes. Rice–crab co-culture increases rice yield and provides economic benefits (Bao et al., 2021). In 2019, a “milky disease” broke out in Chinese mitten crabs cultured in rice fields in Liaoning province. Infected crabs show weakened feeding and movement capabilities. In the later stages of the infection, the hemolymph is completely emulsified, and the whole body turns white until death (Bao et al., 2021; Figure 1). The mortality rate exceeds 20%. Bao et al. (2021) performed pathogen isolation, molecular biological identification, and intramuscular injection experiments, and determined that milky disease is caused by *Metschnikowia bicuspidata* infection. Their results were confirmed by Zhang et al. (2021) and Ma et al. (2022). *M. bicuspidata* is an opportunistic pathogen that can cause serious diseases in different aquatic animals, and harms the aquaculture industry. Moore and Strom (2003) reported that the death rate of Chinook salmon infected with *M. bicuspidata* is as high as 34.5%. From May 2001 to December 2003, *M. bicuspidata* was the dominant pathogen in 6- to 11-month-old *Macrobrachium rosenbergii* giant freshwater prawns (8–12 cm) at water temperatures below 17°C in Taiwan, and the cumulative mortality of prawns during this period was 20–95% (Chen et al., 2007). Recent research on *M. bicuspidata* has focused on the molecular phylogeny, histopathology, and possible control methods of this pathogen (Ma et al., 2022; Sun et al., 2022; Jiang et al., 2022a, 2022b). However, limited information is available about host responses to *M. bicuspidata* (Jiang et al., 2021). Clarifying the host response to pathogens is important in understanding host–pathogen interactions.

With the rapid development of omics technologies, including genomics, transcriptomics, proteomics, metabolomics, and metagenomics, characterization of organisms under different conditions has become a global research hotspot. These technologies have been applied in many fields, such as environmental science, toxicology, immunology, and medical research (Balbi et al., 2021; Ijoma et al., 2021; Islam et al., 2021). The metabolome reflects ongoing life activities during a certain period, and the effects of environmental, physiological, and pathological changes on the body (Liu et al., 2022). Therefore, analyzing the changes in metabolite content and establishing the relationship between metabolites and pathogen invasion can help identify disease biomarkers and elucidate the pathogenesis of diseases (Olive and Sassetti, 2016). Presently, metabolomics is widely used in various fields of medical research, including early diagnosis, personalized treatment, drug target screening, drug efficacy, and toxicity evaluations. Metagenomics has attracted considerable attention in genomics because it allows direct sequencing of microbial communities on a large scale and reveals microbial gene diversity and function. In crustaceans, the intestinal microbiota is closely related to host disease and metabolism. For example, dysregulation of the intestinal microbial composition occurs concurrently with shrimp diseases (Xiong et al., 2015, 2017; Zhu et al., 2016). In addition, a high-dose *Vibrio alginolyticus* attack can dysregulate the intestinal microbial composition of *Portunus trituberculatus* (Xia et al., 2018). Pathogenic bacteria can also change the nutritional availability for the intestinal flora and affect the metabolic functions of the host. Therefore, understanding host–pathogen interactions is important in elucidating how *M. bicuspidata* affects the intestinal microbiota and metabolism of Chinese mitten crabs.

In the present study, we performed metabolomic and metagenomic profiling to create a global survey of differentially expressed metabolites and microbial species and functions between *M. bicuspidata*-infected and healthy crabs. We aimed to understand how *M. bicuspidata* induces gut microbiome and hemolymph metabolomic changes, and how these changes correlate with pathogenesis in *M. bicuspidata*-infected Chinese mitten crabs. Our results provide a theoretical basis for understanding the interactions between fungi and crustaceans, and thus, controlling diseases caused by such interactions.

## Materials and methods

### Experimental design

Approximate 150 Chinese mitten crabs (5.7 ± 0.3 g) were purchased from a farm in Panjin city and transported back to a laboratory at Shenyang Agricultural University. These crabs were temporarily cultured in 300 l glass tanks (temperature 22 ± 0.5°C, pH 7.5 ± 0.2, dissolved oxygen >5 mg/L). They were fed commercial feed (Shenyang Wellhope Aquatic Feed Co., Ltd.) twice daily. The residual food was removed 2 h after feeding, and a quarter of the total water was changed every day. During this temporary culturing period, the hepatopancreas tissue from 10 crabs (randomly selected) was used for PCR detection to ensure that they did not carry *M. bicuspidata*. After 7 days of temporary culturing, the healthy crabs were divided into two groups of 50 individuals each (*M. bicuspidata*-infected and control groups) and then cultured in 300 l glass tanks.

The crabs were fasted 24 h before the injection test to clear their intestinal contents and normalize their metabolic state. Then, the crabs in the treatment tank were injected with *M. bicuspidata* (0.1 ml, 10^7^ CFU) following a method previously described by Bao et al. (2021). The crabs in the control tank were injected with an equal volume of phosphate-buffered saline (PBS). After injection, the water was continuously aerated to ensure sufficient dissolved oxygen, and the crabs were fasted. After 48 h of the injection, 30 crabs were randomly selected from each tank for metabolomic and metagenomic sampling. Under aseptic conditions, the sampled crabs were anesthetized on ice for 5 min, sterilized with 75% alcohol, and then washed with sterilized water to reduce the extent of exotic bacterial contamination. A volume of 200 μl of hemolymph was extracted and mixed with an equal volume of EDTA anticoagulant. The supernatant was collected after centrifugation at 8,000 rpm for 5 min at 4 ° C. Hemolymph samples from five crabs were pooled as one sample to minimize individual differences, immediately frozen in liquid nitrogen, and then transported at –80°C for storage. Each treatment group had six replicates. After the hemolymph was drawn, the crabs were dissected using sterile scissors. The intestinal tract was washed with PBS and then collected in a 1.5 ml centrifuge tube. A total of 10 intestinal tracts were pooled as one sample, quickly frozen in liquid nitrogen, and then stored at −80°C. Each treatment group had three replicates.

### Metabolomic analysis

#### Metabolite extraction

Plasma samples (100 μl) were placed in an EP tube after being thawed at 4°C on ice and then added to 500 μl of extracting solution (methanol: acetonitrile volume ratio = 1:1) containing 2 mg/l L-2-chlorophenylalanine as the internal standard. The samples were ultrasonically treated for 10 min, incubated in ice water at-20°C for 1 h, and then centrifuged at 12,000 rpm for 15 min at 4°C to precipitate the proteins. The supernatant (500 μl) was then removed and dried in a vacuum concentrator. Subsequently, the dried metabolites were redissolved in 160 μl of extraction solution and then centrifuged at 12,000 rpm for 15 min at 4°C after 10 min of ice-water bath ultrasound. The supernatant (120 μl) was carefully placed in a fresh 2 ml LC/MS glass vial. A 10 μl aliquot of the supernatant was obtained from each sample and then pooled as quality control (QC) samples for ultra-high performance liquid chromatography-quadrupole time-of-flight mass spectrometry (UHPLC-QTOF-MS).

#### LC–MS/MS

LC–MS/MS was performed using a UHPLC system (Waters Acquity I-Class PLUS) with a UPLC HSS T3 column (1.8 μm, 2.1 × 100 mm, Waters) coupled to a Waters Xevo G2-XS QTof. The mobile phase comprising 0.1% formic acid aqueous solution (A) and 0.1% formic acid acetonitrile (B) was carried with elution gradient as follows: 0 min, 98% A, 2% B; 0.25 min, 98% A, 2% B; 10.0 min, 2% A, 98% B; 13.0 min, 2% A, 98% B; 13.1 min, 98% A, 2% B; 15.0 min, 98% A, 2% B, which was delivered at 0.4 ml/min. The injection volume was 1 μl. Primary and secondary MS data in MSe mode were collected using a Waters Xevo G2-XS QTof high-resolution mass spectrometer using acquisition software (MassLynx V4.2, Waters). In each data acquisition cycle, dual-channel data acquisition was performed at low and high collision energies of 2 and 10–40 V, respectively, and the scanning frequency was 0.2 s for the mass spectra. The parameters of the ESI ion source were as follows: capillary voltage, 2,000 V (positive ion mode) or −1,500 V (negative ion mode); cone voltage, 30 V; ion source temperature, 150°C; desolvation gas temperature, 500°C; backflush gas flow rate, 50 L/h; and desolventizing gas flow rate, 800 L/h.

#### Data preprocessing and annotation

The raw data collected with MassLynx V4.2 were used for peak extraction, peak alignment, and other data processing operations using the Progenesis QI software, online METLIN database, and Biomarker’s self-built library for identification. The theoretical fragments were discerned, and the mass deviation was within 100 ppm.

#### Data analysis

After normalizing the original peak area information to the total peak area, a follow-up analysis was performed. Principal component analysis (PCA) and orthogonal projections to latent structures discriminant analysis (OPLS-DA) were used to assess the repeatability of the samples within groups and the QC samples. The identified compounds were assessed for classification and pathway information using the Kyoto Encyclopedia of Genes and Genome (KEGG), Human Metabolome Database, and LIPID MAPS Structure Database. According to the grouping information, we calculated and compared the difference multiples and performed *t*-tests to calculate the *p*-value of each compound. The R language package ropls was used to perform OPLS-DA modeling, and permutation tests were performed 200 times to verify the reliability of the model. The variable importance in the projection (VIP) value of the model was calculated using multiple cross-validation. The difference multiples, *p* values, and VIP values of the OPLS-DA model were combined to screen for differential metabolites. The screening criteria were: FC > 1, *p*-value <0.05, and VIP > 1. The different metabolites from KEGG pathway enrichment significance were calculated using the hypergeometric distribution test.

### Metagenomic analysis

#### DNA extraction and illumina high-throughput sequencing

Total genomic DNA was extracted using the PowerSoil® DNA Isolation Kit (MO BIO Laboratories, CA, United States) according to the manufacturer’s instructions. DNA concentration and quality were determined using microspectrophotometry (NanoDrop® ND-2000, NanoDrop Technologies, Wilmington, DE, United States). Metagenomic sequencing was performed using an Illumina HiSeq platform (Biomarker Technologies Corporation, Beijing, China). After quality testing, the genomic DNA was fragmented using ultrasonic interruption. The DNA fragments were purified, end-polished, A-tailed, and then ligated with the full-length adaptor for Illumina sequencing with further PCR amplification to form a sequencing library. The library was first checked by quality inspection and then sequenced using the Illumina sequencing platform at Biomarker Technologies (Qingdao, China).

#### Bioinformatic analysis

The original reads obtained by sequencing were subjected to QC and filtered to obtain clean reads for subsequent bioinformatic analysis. The metagenome was assembled using MEGAHIT software with the default parameters, and contig sequences shorter than 300 bp were discarded (Li et al., 2015). QUAST software was used to evaluate the assembly results (Gurevich et al., 2013). MetaGeneMark software (version 3.26) was used to identify the coding regions in the genome (Zhu et al., 2010). Non-redundant gene sets were constructed using cd-hit software (version 4.6.6; Steinegger and Söding, 2017). The similarity threshold was set to 95%, and the coverage threshold was set to 90%. The protein sequences of non-redundant gene sets were compared with the NR (2019–03) and KEGG (2017–03) databases using the DIAMOND software (version 0.9.24). The threshold was set to an e-value <1e-05. In the case of multiple alignment results (hits), the best alignment result was selected as the annotation of the sequence. Based on the BLAST comparison results with the previous NR database, the genes in the NR database corresponded to the nodes containing functional annotation information in the GO database. Functional genes and species composition were subjected to analysis of similarity using the R vegan package and QIIME, respectively. Wilcoxon rank-sum tests were used for nonparametric tests of the difference between groups of functional genes and species. *p*-values <0.05 were considered statistically significant.

### Integrated metabolomic and metagenomic analyses

Before analysis, the abundances of all species and functional genes were standardized; that is, the abundance of each species and functional gene was divided by the total expression of all species and functional genes in the sample. The Spearman method was used to analyze correlations between the abundance of metabolites with species and functional genes. Results with correlation coefficients >0.90 and *p* values of correlation <0.01 were selected for further analyses. Co-inertia analysis was carried out using the omicade4 package in R.

## Results

### Metabolomic alteration after *Metschnikowia bicuspidate* challenge

#### Multivariate statistical analysis

**Figure 2:**
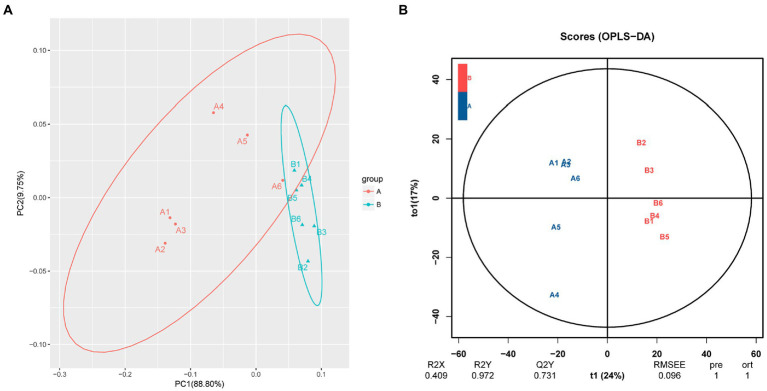
**(A)** Principal component analysis and **(B)** orthogonal projections to latent structures discriminant analysis of all experimental samples used in metabonomic analysis. In the figure, A represents the PBS injection group (control), B represents the *M. bicuspidata* injection group, and A1-A6 and B1-B6 represent 6 replicates.

PCA showed a clear separation in hemolymph metabolite profiles between the *M. bicuspidata*-infected and control groups (Figure 2A). The score scatter plot of the OPLS-DA model, with *R*^2^X = 0.409, *R*^2^Y = 0.972, and *Q*^2^Y = 0.731, showed a distinct separation between the control and *M. bicuspidata*-infected groups (Figure 2B).

#### Differential metabolite analysis

**Figure 3:**
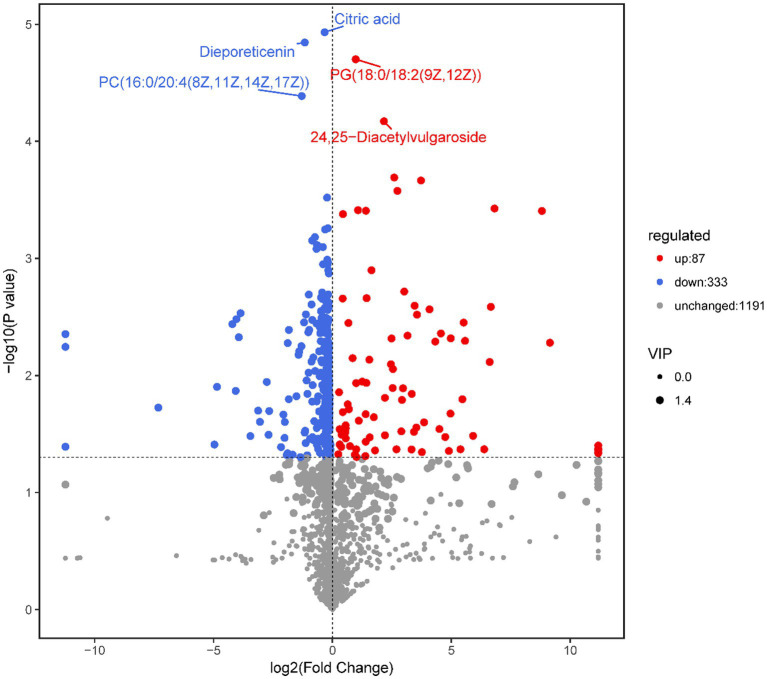
Volcano map of differential metabolites.

Note: Each point in the volcanic map represents a metabolite, the abscissa represents the changes in each substance compared between the groups, the ordinate represents the *p*-value of the *t*-test, and the scatter size represents the variable importance in the projection (VIP) value of the orthogonal projections to latent structures discriminant analysis (OPLS-DA) model.

A total of 1,611 metabolites, including 420 differentially expressed metabolites, were annotated in the *M. bicuspidata*-infected and control groups (Supplementary Table S1). The results of the screened differential metabolites are visualized in the form of a volcano map in all ion modes, as shown in Figure 3. Specifically, 87 differential metabolites were upregulated and 333 were downregulated in the *M. bicuspidata*-infected group compared with the control group. The top 10 upregulated metabolites were lubiprostone, 3-oxopimelyl-CoA, glucosyl passiflorate, 12-hydroxy-7-oxo-8,11,13-abietatrien-18-al, previtamin D3, metanephrine, valtrate, 6-methoxyluteolin 3′-glucoside, rosiglitazone, and 20-COOH-leukotriene E4. The top 10 downregulated metabolites were canesceol, emtricitabine, coriandrone B, phytolaccoside E, 5,6-dihydro-5-fluorouracil (3S,7E,9R)-4,7-megastigmadiene-3,9-diol 9-[apiosyl-(1- > 6)-glucoside], soyasaponin A3, olopatadine, alpha-solanine, and asiaticoside B.

**Figure 4:**
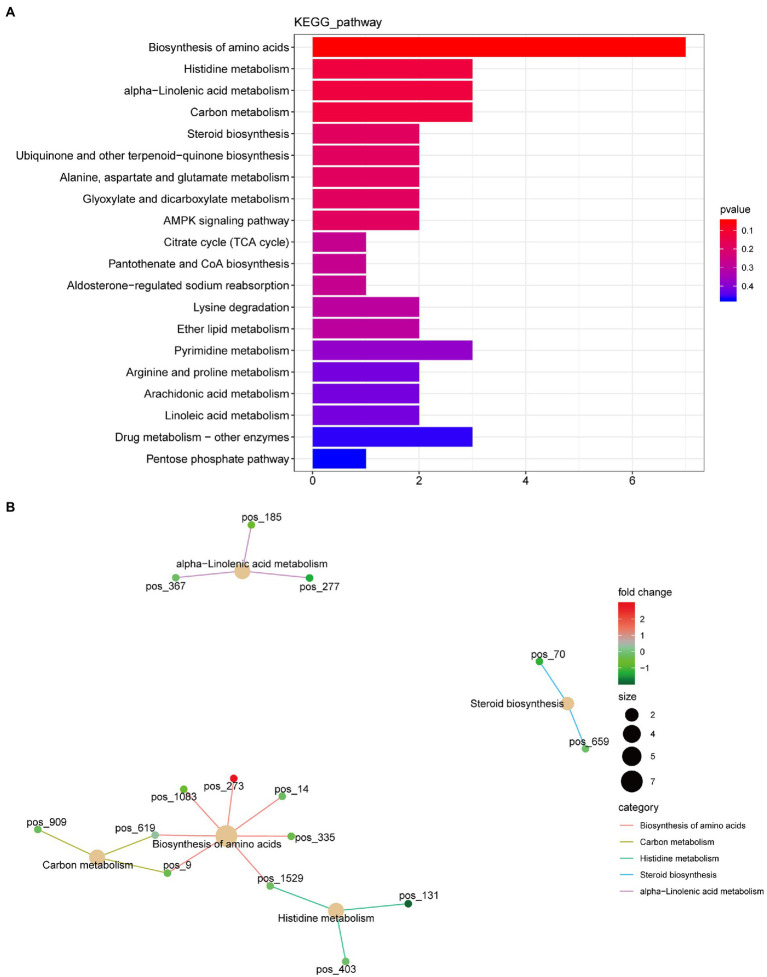
Kyoto Encyclopedia of Genes and Genome (KEGG) function annotation of significantly differential metabolites. **(A)** Classification diagram. The horizontal axis is the number of differential metabolites annotated to the pathway, and the vertical axis is the pathway name. **(B)** Network diagram. Note: the light-yellow nodes in the figure are the pathways, and the small nodes connected to each are the specific metabolites annotated to that pathway. The depth of the color indicates the difference multiple based on the log2 value.

A total of 420 differential metabolites were annotated using the KEGG database. In total, 58 differential metabolic pathways were identified (Supplementary Table S2). The top 20 pathways are shown in Figure 4A, which are mainly enriched in amino acid biosynthesis, histidine metabolism, alpha-linolenic acid metabolism, carbon metabolism, and steroid biosynthesis. Among these pathways, the biosynthesis of amino acids had the highest number of annotated metabolites, including citric acid, L-asparagine, N-acetyl-L-glutamate 5-semialdehyde, lysine, D-sedoheptulose 7-phosphate, S-adenosylhomocysteine, and D-erythro-imidazole-glycerol-phosphate (Figure 4B).

### Metagenomic alteration after *Metschnikowia bicuspidata* challenge

#### Sequencing results and multivariate statistical analysis

**Figure 5:**
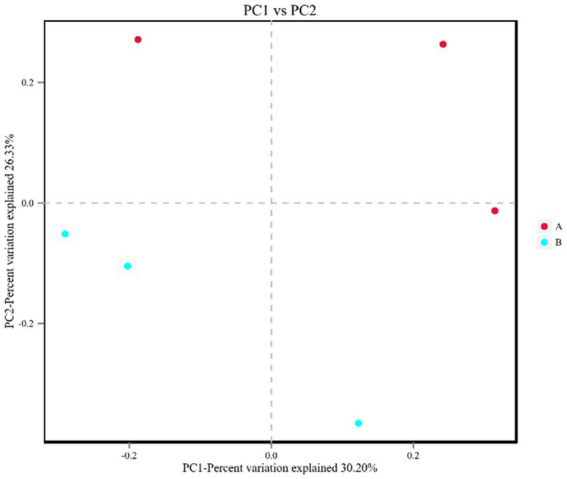
Principal coordinate analysis of all intestinal flora samples. In the figure, A (red dot) represents the PBS injection group (control) and B (blue dot) represents the *M. bicuspidata* injection group. Three red dots represent 3 replicates of PBS injection group, three blue dots represent 3 replicates of *M. bicuspidata* injection group.

After host sequence removal and QC, the sequencing depths of the six samples were approximately 10G. The number of reads in the six samples was between 16,884,470 and 28,120,795. The Q20, Q30, and N50 values were all above 92, 85%, and 770 bp, respectively, indicating that the gene assembly had high integrity and good quality, ensuring the validity of subsequent analyses. The species abundance from the *M. bicuspidata*-infected and control groups was subjected to principal coordinate analysis, and the results are shown in Figure 5. The first two components accounted for 56.53% of the total variation (PC1, 30.20%; PC2, 26.33%).

#### Differentially expressed gene analysis

**Figure 6:**
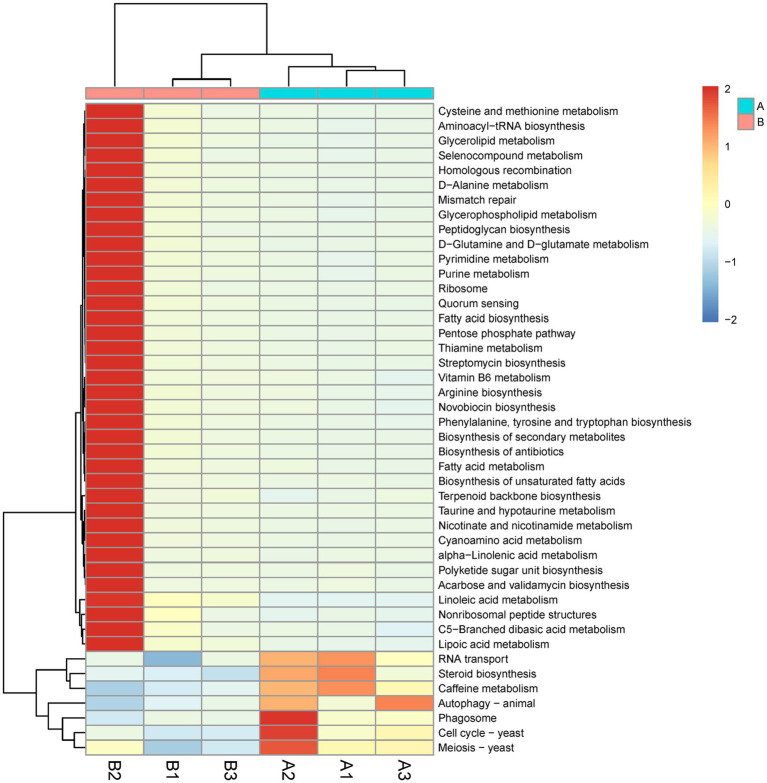
Difference of Kyoto Encyclopedia of Genes and Genome (KEGG) pathway level 3 between the *Metschnikowia bicuspidata*-infected and control groups. In the figure, A represents the PBS injection group (control) and B represents the *M. bicuspidata* injection group. A1-A3 and B1-B3 represent the 3 replicates.

KEGG pathway enrichment analysis of differentially expressed genes revealed 44 differential pathways, of which 37 were upregulated and 7 were downregulated in the *M. bicuspidate* injection group compared to the control group (Figure 6). The top three upregulated pathways were cysteine and methionine metabolism, aminoacyl-tRNA biosynthesis, and glycerolipid metabolism. The top three downregulated pathways were RNA transport, steroid biosynthesis, and caffeine metabolism.

#### Analysis of differences in species composition

**Figure 7:**
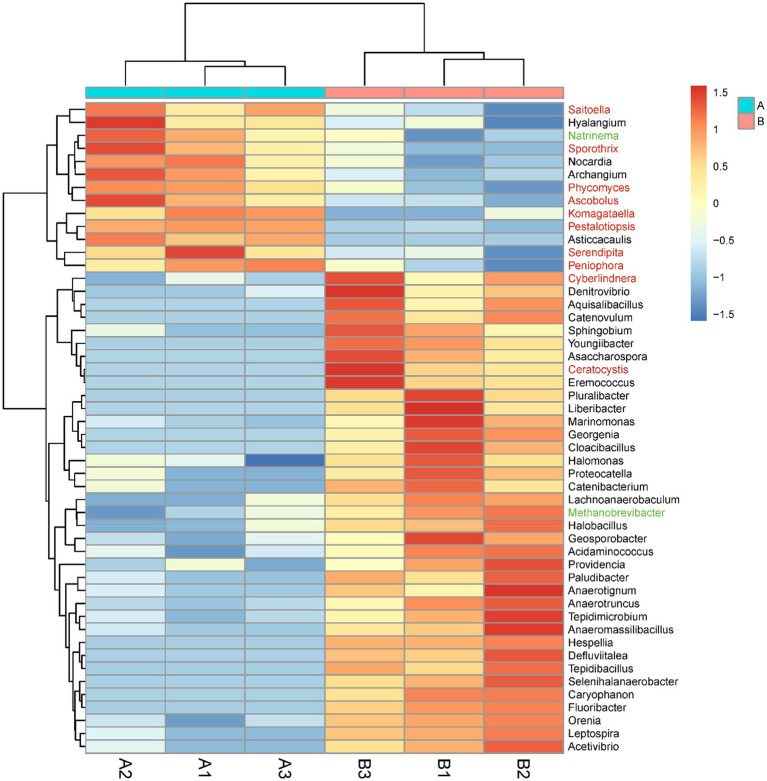
Genus-level abundance difference between the *Metschnikowia bicuspidata*-infected (B1, B2, B3) and control (A1, A2, A3) groups. A represents the PBS injection group (control) and B represents the *M. bicuspidata* injection group. The red font represents Fungi, the black font represents Bacteria, and the green font represents Archaea.

The abundances of eight orders of microorganisms significantly changed in the *M. bicuspidata*-infected group compared with the control group (Supplementary Figure S1). Specifically, the abundances of Methanobacteriales, Rhodocyclales, Acidaminococcales, and Leptospirales significantly increased, whereas those of Frankiales, Caldilineales, Corynebacteriales, and Sebacinales significantly decreased. The abundances of 50 genera of microorganisms significantly changed in the *M. bicuspidata*-infected group compared with the control group (Figure 7). The abundances of 37 genera, including *Cyberlindnera*, *Denitrovibrio*, and *Aquisalibacillus*, significantly increased, whereas those of 13 genera, including *Saitoella*, *Hyalangium*, and *Natrinema*, significantly decreased (Supplementary Table S3).

### Combined metabolomic and metagenomic analyses

**Figure 8:**
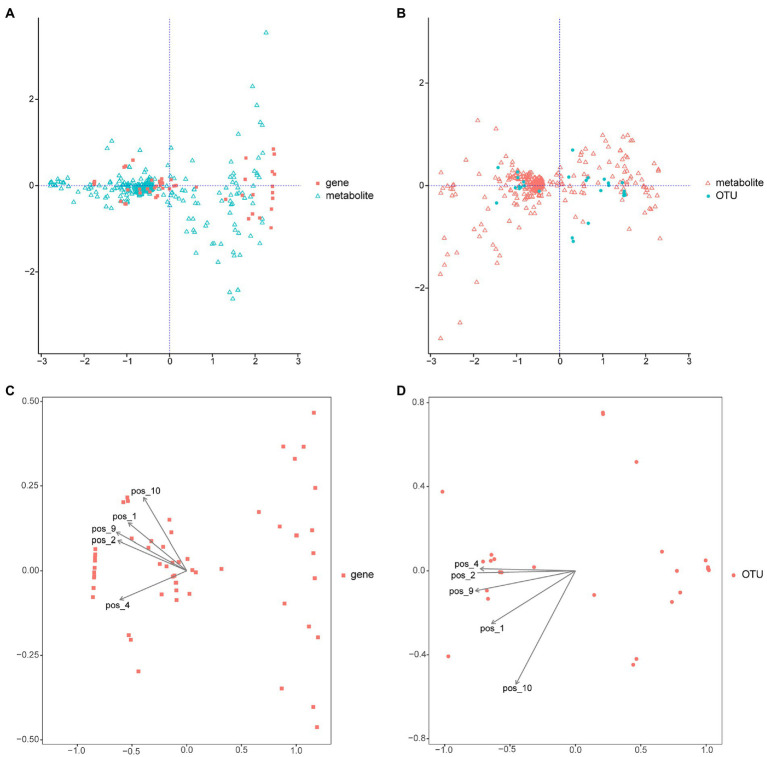
Integrated metabolomic and metagenomic analyses. **(A)** Co-inertia analysis of gene function and different metabolites. **(B)** Co-inertia analysis of operational taxonomic units and different metabolites. **(C)** Constrained correspondence analysis of gene functions and different metabolites. **(D)** Constrained correspondence analysis of operational taxonomic units and different metabolites. OUT is operational taxonomic units; pos_1 is epsilon-Captrolactone, pos_2 is N-formylglycine, pos_4 is monoethyl malonate, pos_9 is citric acid, and pos_10 is 6-hydroxydopamine hydrochloride.

Integrated metabolomic and metagenomic analyses were conducted to obtain comprehensive insights into the changes in metabolic pathways during *M. bicuspidata* infection (Figures 8A,B). The metabolites epsilon-caprolactone, N-formylglycine, monoethyl malonate, citric acid, and 6-hydroxydopamine hydrochloride were closely related to the population structure and gene function of different intestinal microorganisms (Figures 8C,D). The results of KEGG enrichment analysis revealed that only the caffeine metabolism pathway was significantly enriched.

## Discussion

Milky disease caused by *M. bicuspidata* can cause serious economic losses for the *E. sinensis*, *Portunus trituberculatus*, *Palaemonetes sinensis*, and *Macrobrachium rosenbergii* aquaculture industries (Chen et al., 2007; Wang et al., 2007; Bao et al., 2021; Cao et al., 2022). However, little is known about the host response mechanisms to *M. bicuspidata* infection. In the present study, the differential hemolymph metabolites between *M. bicuspidata*-infected crabs and control crabs were compared using metabolomics. PCA clearly distinguished the hemolymph metabolite spectra between the control and *M. bicuspidata*-infected groups. The results showed many changes surrounding metabolites and pathways involved in amino acid metabolism.

Amino acid biosynthesis is the basis of protein biosynthesis and affects many biological reactions (Lengyel and Söll, 1969; Ibba et al., 2001). *Vibrio parahaemolyticus* infection in *Scylla paramamosain* mud crabs can significantly alter their amino acid biosynthesis (Kong et al., 2020). In the present study, the amounts of metabolites [citric acid and L-asparagine (Asn)] involved in amino acid biosynthesis were generally lower in *M. bicuspidata*-infected crabs than in healthy crabs. Citric acid is the key metabolite in the tricarboxylic acid (TCA) cycle (Akram, 2014). Asn can be converted into asparagine, which can form oxaloacetic acid *via* transamination to enter the TCA cycle (Cooney et al., 1970). The TCA cycle is the hub that connects carbohydrate, fat, and amino acid metabolism in aerobic organisms (Kumar and Dubey, 2019). The results of the present study indicate that *M. bicuspidata* affected the TCA cycle by downregulating citric acid and Asn levels to decrease sugar, fat, and amino acid metabolism and subsequently, the energy supply efficiency of Chinese mitten crabs. Similarly, a previous study showed that *V. parahaemolyticus* infection reduces the amounts of TCA cycle intermediates (Kong et al., 2020).

Similarly, the lysine and histidine metabolic pathways were significantly affected by *M. bicuspidata* infection. Lysine is an essential amino acid in aquatic animals. Lysine deficiencies can hinder biological growth, reduce feed conversion and protein deposition rates, and weaken the free radical scavenging abilities of aquatic animals (Forster and Ogata, 1998; Luo et al., 2006; Li et al., 2016, 2019). Histidine is also an amino acid involved in many metabolic functions, including histamine production, osmoregulation, and energy production (Sakata et al., 1997; Khan, 2018). In the present study, the differential metabolites involved in lysine and histidine metabolism were significantly downregulated, suggesting that *M. bicuspidata* infection affects protein synthesis, histamine metabolism, and growth and osmotic pressure regulation in Chinese mitten crabs.

Linolenic acid, arachidonic acid, and linoleic acid are essential fatty acids that play important roles in the physiological, biochemical, and immune functions of aquatic animals (Glencross and Smith, 2001; El-Husseiny et al., 2010). In the present study, the PC (16:0/16:0), PC [16:0/20:4 (8Z, 11z, 14z, 17Z)], and JA contents significantly decreased after *M. bicuspidata* infection, indicating that the linolenic, arachidonic, and linoleic acid metabolic pathways were significantly inhibited, which affected the growth and immune function of Chinese mitten crabs. The AMP-activated protein kinase (AMPK) signaling pathway, which is involved in growth and immune function, was also significantly inhibited. AMPK is a central regulator of cell energy homeostasis and plays a key role in controlling cell growth and other processes, including autophagy and polarity (Causey et al., 2019). Jiang et al. (2021) found that the expression of several hemolymph immune proteins changed after *M. bicuspidata* infection. We obtained similar results, validating the hypothesis that Chinese mitten crabs generate a strong immune response to *M. bicuspidata* infection. This phenomenon was also observed in a study on *Spiroplasma eriocheiris* infection in Chinese mitten crabs (Meng et al., 2014).

Among the different metabolites in this study, the number of upregulated metabolites was significantly lower than that of downregulated metabolites. Among the upregulated metabolites, the pathways of steroid hormone biosynthesis and aldosterone synthesis and secretion were significantly enriched. Steroid hormones are major regulators of physiological and pathological processes (Benagiano et al., 2019; Yang et al., 2021). Aldosterone enhances ion and water reabsorption by the kidneys (Salyer et al., 2013). When the circulating blood volume decreases, aldosterone secretion increases, enhancing the reabsorption of sodium and water to maintain homeostasis (Schrier and Regal, 1972). The significant increase in aldosterone levels found in the present study may also be related to the reduction in the number of hemocytes (Chen et al., 2007). After infecting Chinese mitten crabs, yeast pathogens like *M. bicuspidata* rapidly proliferate in the hemolymph, and hemocytes reduce and eventually lose the ability to agglutinate, appearing milky white (Jiang et al., 2021).

The intestinal microflora plays an important role in the metabolism, immunity, and homeostasis of crustaceans (Huang et al., 2018; Qi et al., 2021). Dramatic changes in living environments lead to imbalances in host intestinal microflora, which seriously affects development, nutrition, immunity, and disease resistance (Sun et al., 2018). In the present study, *M. bicuspidata* infection altered the intestinal microflora of Chinese mitten crabs, and specifically, the abundances of Methanobacteriales, Rhodocyclales, Acidaminococcales, and Leptospirales significantly increased. Bacteria belonging to Methanobacteriales are strict anaerobes that grow *via* H_2_ oxidation (Bonin and Boone, 2006). They are widely distributed in nature and exist in anaerobic habitats, such as aquatic sediment, soil, anaerobic sewage digesters, and animal gastrointestinal tracts. Similarly, Rhodocyclales and Acidaminococcales are also adapted for anaerobic environments (Rands et al., 2019; Wang et al., 2020). These findings indicated that *M. bicuspidata* infection significantly increased the abundance of anaerobic bacteria in Chinese mitten crabs.

At the genus level, 37 genera increased significantly, and 21 genera belonged to the phylum Firmicutes, of which 11 genera belonged to the order Eubacteriales, including *Hespellia*, *Acetivibrio*, *Lachnoanaerobaculum*, *Youngiibacter*, *Defluviitalea*, *Proteocatella*, *Anaeromassilibacillus*, *Asaccharospora*, *Anaerotruncus*, *Geosporobacter*, and *Anaerotignum*. These bacteria mainly perform anaerobic respiration, and grow better under anaerobic conditions than under aerobic conditions. These bacteria lack a complete metabolic enzyme system, and carry out energy metabolism *via* anaerobic fermentation (Lowe et al., 1993). Most bacterial pathogens in aquatic animals are aerobic gram-negative bacilli (Joshi et al., 2014; Cao et al., 2015). A few anaerobic bacteria can become pathogenic in aquatic animals. However, these findings may also be due to a knowledge gap. Most of the 23 recorded obligatory anaerobes are human, mammal, bird, and/or reptile pathogens (Abdelsalam, 2017). Among the anaerobic bacteria with increased abundance in the present study, *Lachnoanaerobaculum*, *Anaeromassilibacillus*, and *Youngiibacter* can cause disease in humans. *Lachnoanaerobaculum* was first isolated from the jejunal mucosa of a child with celiac disease (Moore et al., 2022). Ida et al. (2022) found that *Lachnoanaerobaculum* could cause human bacteremia. *Youngiibacter* has been correlated with astrocyte activation in some neurological disorders (e.g., autism spectrum disorder), and *Anaeromassilibacillus* has been isolated from the gut of patients with kwashiorkor (Guilhot et al., 2017; Tomova et al., 2020). Considering the importance of anaerobic bacteria in veterinary and public health, aquatic health experts should pay special attention to anaerobic bacteria. Investigating the types of anaerobes isolated from aquatic animals may help clarify the role of anaerobes in aquatic animal disease outbreaks. In the present study, 13 genera of microorganisms decreased significantly after *M. bicuspidata* infection, with most belonging to the fungal phylum Ascomycota, including *Ascobolus*, *Komagataella*, *Pestalotiopsis*, *Saitoella*, and *Sporothrix*. Ascomycota is an important component in the intestinal tract of Chinese mitten crabs (Xu et al., 2021). In the present study, a large number of ascomycetes were found in the intestinal tract of the control group. However, *M. bicuspidata* reduced the number of these fungi. This result may be due to the ability of *M. bicuspidata* to inhibit the growth of similar species, such as ascomycetes, with proximal niches (Iacob et al., 2019).

From the perspective of the functions of intestinal flora, many metabolic and synthetic pathways were significantly upregulated, whereas immunity-related pathways were significantly downregulated. This result indicated that *M. bicuspidata* infection increased the nutrient demand of microorganisms in the gut, which consequently enhanced the metabolism and synthesis of amino acids, fatty acids, glycolipids, cystine, and methionine. The massive proliferation of these microorganisms reduces immune-related pathways, such as phagosome formation and autophagy in the intestine, which may lead to immune evasion by some opportunistic pathogens and increased risk of mixed infections (Aranguren et al., 2017).

Integrated metabolomic and metagenomic analyses identified 55 differentially expressed genes and 28 operational taxonomic units. These genes and operational taxonomic units had good correlations with the 420 differential metabolites identified in the metabonomic analysis. Thus, *M. bicuspidata* infection altered the intestinal microbial composition and metabolism of Chinese mitten crabs, which also had effects on each other, and consequently affected the metabolism, growth, immunity, and other aspects of the crabs. KEGG pathway analysis of the differential metabolites and functional genes showed that only the caffeine metabolism pathway was significantly enriched. Caffeine is decomposed in the hepatopancreas to produce three primary metabolites: paraxanthine, theobromine, and theophylline (Lelo et al., 1986). After absorption, it is distributed throughout the body. In the present study, the theophylline content and xanthine dehydrogenase gene expression in the caffeine metabolic pathway significantly changed. Theophylline and xanthine exert stimulant and bronchiectasis-promoting effects on humans (Kennedy, 2021), but their effects on aquatic animals are unclear. Therefore, their significance after *M. bicuspidata* infection requires further investigation.

In conclusion, *M. bicuspidata* infection altered the metabolism and intestinal microflora of Chinese mitten crabs. It significantly inhibited amino acid biosynthesis, the TCA cycle, lysine and histidine metabolism, and linolenic, arachidonic, and linoleic acid metabolism. It also increased the abundance of anaerobic bacteria in the intestine while significantly inhibiting immune-related metabolic pathways. Consequently, *M. bicuspidata* infection affected the growth and immunity of Chinese mitten crabs.

## Data availability statement

The datasets presented in this study can be found in online repositories. The names of the repository/repositories and accession number(s) can be found at: NCBI, PRJNA865396. https://dataview.ncbi.nlm.nih.gov/object/PRJNA865396?reviewer=16j500f940cii066tr3df5ndm4.

## Ethics statement

The animal study was reviewed and approved by Animal Experiments Ethics Committee of Shenyang Agricultural University.

## Author contributions

HJ and QC: conceived and designed the project. JB, HJ, GC, and YX: prepared the samples and conducted the bioinformatics analysis. HJ, JB, XL, and QC: wrote the manuscript. All authors contributed to the article and approved the submitted version.

## Funding

This work was supported by China Agriculture Research System of MOF and MARA (CARS-48); Liaoning province Department of Education fund item (LSNQN202002); Liaoning province “The Open Competition Mechanism to Select the Best Candidates” Project (2021JH1/10400040); and Shenyang Science and Technology Mission Project (21–116–3-38).

## Conflict of interest

The authors declare that the research was conducted in the absence of any commercial or financial relationships that could be construed as a potential conflict of interest.

## Publisher’s note

All claims expressed in this article are solely those of the authors and do not necessarily represent those of their affiliated organizations, or those of the publisher, the editors and the reviewers. Any product that may be evaluated in this article, or claim that may be made by its manufacturer, is not guaranteed or endorsed by the publisher.

## Supplementary material

The Supplementary material for this article can be found online at: https: //www.frontiersin.org/articles/10.3389/fmicb.2022.990737/full#supplementary-material
