# Supplementary figures and images for: Metabolomic and metagenomic analyses of the Chinese mitten crab Eriocheir sinensis after challenge with Metschnikowia bicuspidata
Source: Front Microbiol. 2022 Sep 23;13:990737. doi: 10.3389/fmicb.2022.990737 (PMC9538530; doi:10.3389/fmicb.2022.990737)

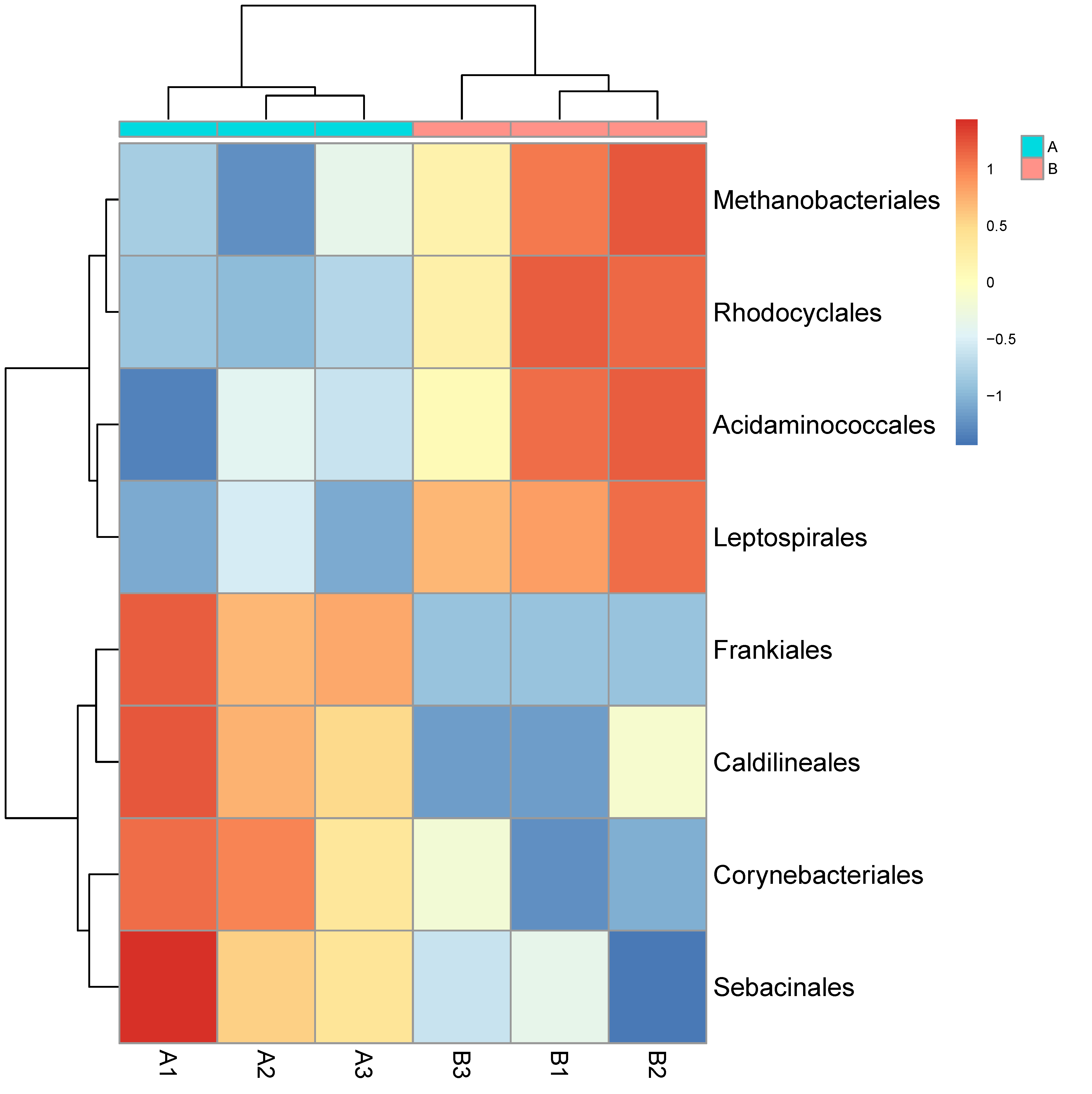

Supplement: Supplementary file 4 [file Image_1.TIFF]
